# Prehospital providers’ perspectives for clinical practice guideline implementation and dissemination: Strengthening guideline uptake in South Africa

**DOI:** 10.1371/journal.pone.0219761

**Published:** 2019-07-22

**Authors:** Michael McCaul, Lynn Hendricks, Raveen Naidoo

**Affiliations:** 1 Centre for Evidence-based Health Care, Division of Epidemiology and Biostatistics, Stellenbosch University, Cape Town, South Africa; 2 Director Emergency Medical Services & Disaster Medicine, National Department of Health, Johannesburg, South Africa; Medical University Graz, AUSTRIA

## Abstract

**Background:**

In 2016 the first African emergency care clinical practice guideline (CPG) was developed for national uptake in the prehospital sector in South Africa, with implementation starting in 2018. Comprehensive uptake of CPGs post development is not a given, as this requires effective and efficient dissemination and implementation strategies that take into account the perceptions, barriers and facilitators of the local end-users. This study aimed to identify prehospital end-users’ perceptions of the emergency care guidelines, including barriers and facilitators for national decision makers, to strengthen CPG uptake in South Africa.

**Methods:**

Our study employed a descriptive qualitative research design, including nine focus groups with 56 operational emergency care providers across four major provinces in South Africa. Data was analysed using thematic analysis in ATLAS.ti. Ethics approval was provided by Stellenbosch University.

**Results:**

Themes related to provider perceptions, expectations and guideline uptake emerging from the data was unofficial and unclear communication, broadening versus limiting guideline expectations, conflicted personal reactions and spreading the word. Challenges to dissemination and implementation included poor communication, changes to scope of practice, and limited capacity to upskill existing providers. Facilitators included using technology for end-user documents, local champions to support change, establishing online and modular training, and implementation by independent decision makers.

**Conclusion:**

This study provides an overview of the perceptions of operational emergency care providers and how their experiences of hearing about and engaging with the guidelines, in their industry, can contribute to the dissemination, implementation and uptake of emergency care guidelines. In order to disseminate and implement an emergency care CPG, decision makers must take into account the perceptions, barriers, and facilitators of local end-users.

## Introduction and context

The Health Professions Council of South Africa: Professional Board of Emergency Care (HPCSA PBEC) guides and regulates the emergency care profession regarding registration, education, training, and professional conduct, as per the Health Professions Act of 1974. To date, emergency care clinical practice has been guided by protocols, documents providing clinical practice instructions, last revised in 2006 and 2009 [1–3]. With unclear and outdated evidence underpinning the protocols, the PBEC initiated the revision of the protocols in August 2015 [4]. The African Federation for Emergency Medicine (AFEM), collaborating with researchers and emergency care specialists, was awarded the bid to revise and reformulate the protocols using best evidence in late 2015.

Adaptive guideline development methods were used, where existing up-to-date high-quality clinical practice guidelines (CPGs) were synthesised, instead of primary evidence, and either adopted, adapted or contextualised to the local setting leading to the production of the first evidence-based clinical practice guideline for the emergency care profession in Africa [5]. The CPG culminated in a document with over 1000 recommendations for South African emergency care clinical practice, aligned to local contextual factors. The CPGs represent a transition from skills-driven (protocolised) practice underpinned by expert opinion, to practice that is informed by the best available evidence. Further details around guideline methods and challenges have been reported elsewhere [6]. Since the first submission of the CPG to the HPCSA PBEC in middle 2016 [7], the CPG has undergone public comment, including input from the National Department of Health (NDoH), higher education institutions, other regulatory bodies, and most importantly the guideline end-users, and has officially been ratified and released for implementation in December 2018 [8].

The CPG has been met with fierce resistance because the guideline recommendations and inferred updated scope of practice for providers has vast implications for emergency care service delivery, training, and by extension, curriculum alignment for a total of seven different qualification registries, affecting approximately 70 000 registered providers [9]. Some implications are considered positive (e.g. access to effective treatments previously unavailable), others are considered negative (e.g. narrowing the scope of practice for some providers); overall however, the new emergency care guidelines have brought change and discourse to prehospital care in South Africa. The South African Emergency Medical Services (EMS) prehospital qualification framework is complex, with prehospital training ranging from three weeks to four years (S1 Table).

The current status quo is a mix of different qualifications ranging from basic life support (BLS) to highly trained practitioners with a variety of skills, knowledge and tools to perform advance emergency care and rescue. The majority of EMS providers have a 4-week (BLS) and 3-month intermediate life support (ILS) qualifications. Currently the 4-week, 9 month and 2 year National Certificate courses have been phased out, the 3-month and 3 year National Diploma courses are being phased out as industry transitions to professionalise emergency care providers away from skills based short course training programs. Considering this, the successful dissemination and implementation of the guidelines have been referred to as the ‘biggest challenge yet’ facing South African emergency care [5] as emergency care policy, curriculum, approval of new medicines, training of providers and industry responds to change.

Globally, an important intervention for maximising the clinical impact of CPGs is an assessment of local barriers and perceptions of the target users [10], for which evidence is currently lacking for prehospital care in resource-constrained settings [11,12]. Decision makers, including industry service providers, the National Department of Health, regulators, and training institutions, need to be aware of the perceptions, experiences, challenges and solutions expressed by prehospital providers for guideline implementation and dissemination to strengthen guideline uptake and have lasting impact on patient outcomes. In order to strengthen guideline implementation, we sought to understand prehospital providers’ experiences with guidelines and identify challenges and solutions to guideline implementation and dissemination.

## Methods

Our study employed a qualitative descriptive research design. We conducted focus groups with operational emergency care providers across four major provinces in South Africa. The data were thematically analysed, to identify the main perceptions and experiences of individuals or groups of individuals at a particular point in time [7,13–15]. Participants provided written informed consent. The research was approved by the Stellenbosch University Research Ethics Committee (N17/02/018).

### Context

It is important that the below findings and processes are read within the temporal context of the study. Focus groups were conducted in early 2017 during which the CPGs had already been submitted to the PBEC (mid-2016). The pre-release communication from the PBEC to providers was on March 2016 [16], reporting on the progress and scope of practice review process, followed by an unintended leak of the CPGs. The PBEC then formally released a draft version of the CPGs to prehospital providers and educators for comment in October 2016 for initial comment, for implementation by education providers in June 2017 [17] followed by the official ratified version and communication for implementation in December 2018 [18].

### Participants

The research was conducted across four major provinces in South Africa, namely the Western Cape, Gauteng, Eastern Cape and Kwa-Zulu Natal, representing the heterogeneous nature of the prehospital workforce distribution. Purposive snowball sampling was used to include 56 operational prehospital providers across the provinces. We purposefully invited more public providers than privately employed providers to consider the South African EMS workforce distribution. An equal mix of urban (providers working exclusively in minor city areas) versus rural providers was sought. During the invitation period, we identified potential participants by contacting prehospital societies, local colleges, universities, and employers. Participants needed to have an active registration with the HPCSA to be eligible. Potential participants were contacted telephonically, introduced to the study and study team and invited to participate in a focus group. They were asked to suggest a colleague to invite to the study that purposefully fit the intended distribution of qualifications and geographic settings (urban vs rural). Focus group participation was confirmed via email, detailing focus group venue and time, informed consent, and study details that was discussed telephonically. They were informed that the study was not being conducted on behalf of the HPCSA nor the National Department of Health (NDoH) but as an independent research project from Stellenbosch University, as the investigators were concerned about potential animosity any participants might display towards certain stakeholders.

### Data collection

Focus groups were held in boardroom or classroom settings across provinces at local universities or colleges in mid-2017. Only the participants and researchers (MM and LH) were present during focus groups. Each province (except for KwaZulu-Natal) had two focus groups, for private and public providers separately. Investigators conducted three focus groups (two public, one private) in KwaZulu-Natal as in the public sector there was a larger advanced life support interest (BTech/BSc, ECT, CCA and NDip) on the day compared to providers with junior qualifications (AEA and BAA) (S1 Table). At focus groups an informal conversational atmosphere was promoted by seating chairs in a circular arrangement to facilitate informal discussion, talking to participants as they arrived and sharing refreshments while getting to know one another. Providers were asked to provide written informed consent, followed by a didactic informal presentation by the investigators describing the project details (research team, objectives, project background and process), history and process of the CPGs to date, followed by a question and answer session. MM, a male ECP and the principal investigator, lead the focus groups, and was supported by LH, a female qualitative research consultant. Focus groups lasted approximately 2–3 hours with an average size of seven participants per focus group.

### Data collection instruments

All authors were involved in the design of the focus group interview schedule and the schedule was reviewed by representatives from NDoH and the HPCSA PBEC to facilitate knowledge translation of results into policy and practice. The interview schedule was divided into three sections: guideline perceptions and expectations, guideline dissemination, and implementation. Questions such as ‘When you received the guidelines for the first time, what did you expect to see?’ and ‘What are your thoughts on how you think the guidelines can be implemented?’. Focus groups were recorded and transcribed verbatim by an independent contractor. A summary of the findings, as an infogram, was provided to all participants via email for comment and to do date no comments have been received.

### Data analysis

The data was analysed through a thematic analysis, using the phases described by Braun and Clarke [15]. These include i) familiarising oneself with the data ii) generating initial codes iii) searching for and reviewing themes. Two researchers (MM and LH) coded 7 transcripts through line-by-line reading with the aid of Atlas.ti v7 initial codes. These codes were then categorised into potential themes related to the study objectives and guided by the interview schedule across the entire dataset, gathering all data relevant to each potential theme. We reviewed themes and codes by generating a thematic map of the analysis. The project team met regularly to clarify and define emerging themes.

### Reflexivity

Throughout the stages of the study, we attempted to adhere to the methodological principle of reflexivity [19]. This involved all researchers being aware of, and critically examining, their positioning and assumptions. Various steps were also taken to minimise how these might inappropriately influence the research process and outcomes.

The principle investigator (MM) is an emergency care practitioner (paramedic) and was involved in developing the original EMS CPGs for the HPCSA PBEC as a methodologist in the AFEM core guideline panel. MM was an operational paramedic for approximately 4 years in the private EMS sector before becoming a researcher at Stellenbosch University. In proposal development and data analysis MM drew from operational experience and knowledge of the South African EMS systems to strengthen the contextual framework of the study results. Having previously been an operational paramedic, MM was able to bridge the perceived hierarchy gap between researchers and paramedics during focus group discussions, especially within the private sector but less so in the public sector. As a methodologist and guideline panel member for the AFEM CPGs, MM acknowledged his bias in favour of the guidelines. He objectively distanced himself from influencing conversation (directly or indirectly), perceived misperceptions, and commentary around the guideline development during focus group interviews by handing over to the qualitative researcher (LH) during the focus group discussion. During coding, analysis, and report writing, MM worked together with LH to reflect on participants’ insights in the context of the current guideline context. MM drew on his operational experience as a paramedic and LH drew on her experiences as a qualitative researcher to understand each individual provider’s context. RN used his experience as a decision maker to provide insight into understanding the implementation and dissemination challenges within the emergency care system.

### Trustworthiness

In this study the authors sought to ensure that the research process was trustworthy, so that the findings could be considered a credible reflection of reality [19]. Several measures were taken to establish credibility, dependability, confirmability and transferability, where possible. These included peer scrutiny of the project and data, description of study context, debriefing sessions, iterative questioning, purposeful sampling, rich use of quotations from participants and admission of research beliefs and assumptions.

## Findings

56 people participated in the study, across 7 focus groups, the majority of whom were male, with a relatively equal balance regarding location. Participants came from a number of different organisations, with varied levels of operational experience and educational background. Characteristics of included participants can be seen in Table 1.

**Table 1:** Characteristics of focus group participants.

Characteristic	
**Age (years), mean (SD)**	36 (7.4)
**Gender, n (%)**	
- Male	44 (79)
- Female	12 (20)
**Qualification, n (%)**	
- BTech/BSc	13 (23)
- NDip	2 (4)
- ECT	7 (13)
- CCA	10 (18)
- AEA	15 (26)
- BAA	9 (16)
**Employer, n (%)**	
- Private	19 (34)
- Public	37 (66)
**Location, n (%)**	
- Urban	32 (57)
- Rural	24 (43)

See S1 Table for abbreviations.

During focus groups, participants were already exposed to the CPGs. The below themes emerged from the data in response to questions around how they first heard about the guidelines, their expectations of the guideline and challenges and solutions to guideline implementation and dissemination. Themes from the data were:

- Unofficial and poor communication
- Broadening versus limiting guideline expectations
- Conflicted personal reactions
- Spreading the word
- Challenges and opportunities for dissemination and implementation

### Unofficial and poor communication

We asked participants when and how they first heard about the CPGs. Here responses varied including hearing about the guidelines via informal channels, receiving documents across social media, and word of mouth; while some providers were unaware of any CPGs.

The guidelines was described as an unofficial release, containing an incomplete version of the original CPGs (excluding the methods), a large document with clinical practice recommendations, and a checkbox list of new capabilities and skills for all prehospital providers. These documents were disseminated across social media (e.g. Facebook), communication platforms (i.e. WhatsApp), email, or word of mouth while providers worked shifts:

*“It wasn’t anything that came from HPCSA*, *it wasn’t anything that was sent to us via formal channels*. *It was literally social media…”* (CCA, private sector).

*“I basically just heard as we were sitting one evening on shift having coffee…*, *but somebody got the document*, *two hundred and I don’t know how many pages…*, *it’s just bits and pieces of the thing because it’s quite a huge document and the person didn’t read everything”* (BAA, private sector).

However, some were not aware of the new CPGs or any decision to review the old protocols.

*“To be honest*, *I think the first time I hear about it*, *because I’m not a social media person”* (BAA, public sector, rural area).

### Broadening vs limiting guideline expectations

We asked participants what they expected to see from the new CPGs. These expectations varied between broadening and limiting guideline sub-themes.

Broadening expectations included the expectation of increased scope of practice and training opportunities, to advance clinical freedom, while limiting expectations included autocratic decisions from regulators and a perceived negative agenda.

Providers expected “*to extend our scope of practice*” (BAA, public sector), with redundant skills, practices or pharmacopoeia to be phased out. Providers expected these changes to be accompanied by training opportunities, specifically for providers within the existing EMS by *“incorporating this training for the old guys”* (CCA, public sector).

Providers expected the CPGs to *“broaden the views for us to be able to think out of the box*” (ECT, public sector), expressing the need to move away from the ‘tick box’ approach of allocating scopes of practice and placing providers in hierarchical silos. Some expressed this notion as “*using your own clinical judgment”* (CCA , private sector) or *“introducing an element of choice in terms of decision-making and patient’s best interest…”* (ECP, private sector).

However, limiting expectations included concerns that the CPGs *“has potential to drive a certain perceived agenda within the industry”* (CCA, private provider), and concerns around autocratic communication and decision style from regulators:

*“The word specifically is bulldozed*, *that this is going to be bulldozed through and forced down our throats*, *whether you agree with it or not”* (AEA, private sector).

### Conflicted personal reactions

Providers reacted variedly to the guidelines, often with conflicting views. Some providers reacted with excitement, some felt their competence and worth as advanced short course paramedics was being questioned, while others mentioned that resource constraints limit providers from retraining at universities.

Some providers were content with the proposed guidelines, specifically the proposed ‘Appendix A’ section *“where 90% of them went first shot”* (ECP, private sector) as very few were concerned with reading the large CPGs document due to the predominant interest in scope of practice changes. Junior providers reacted with excitement to see an increase in their scope of practice while advanced providers were excited to see a focus shift to guidance that is evidence-based with an emphasis on previously neglected clinical topics:

*“I was very excited obviously to see the BLS going to get more drugs and ILS get more drugs”* (BAA, public sector).

*“We’ve got better focus on areas that have been traditionally horribly neglected such as the management of the obstetric patient”* (ECP, private sector).

*“Everybody’s for it… finally it’s actually more evidence-based than what it has ever been”* (CAA, private sector).

However, some providers had specific skills removed off their existing scope of practice, like drug facilitated endotracheal intubation (ETI) from CCAs, historically viewed as an exclusive advanced life support skill. Paramedics expressed this as a *“slap in my face”* (CCA, public sector) expressing anger, shock, disappointment, and fear, and felt that the further emphasis on scopes of practice establishes hierarchical silos and division across providers. Paramedics strongly identified with the skill of endotracheal intubation; paramedics see endotracheal intubation as part of their identity and self-worth: “*What is my qualification then actually worth*?” (re removal of ETI, CCA, private sector).

This is a sensitive point for CCAs specifically, as they see themselves as the original advanced life support providers. Advanced short course paramedics feel that their competency as emergency care providers has been questioned; they feel sidelined, abandoned and literally *“scrapped off the register”* (CCA, public sector).

*“NDips and CCA’s are the only ones that are actually registered as paramedics with the HPCSA*, *but now we are not paramedics anymore…*” (re removal of ETI, CCA, private sector).

Some emergency care providers feel trapped or “*“being stuck”* (BAA, private sector) in the current educational framework, especially with the introduction of the new CPGs and scopes of practice. Providers are apprehensive about how they are going to be upskilled and cost.

*“Probably all of us need to go to university because it’s too much information and you can’t just start working and using the guidelines”* (ECT, public sector).

Providers feel they are left with no choice but to *“resign*, *go to university for four years and get your degree”* (CCA, public sector) in order to upskill. However, many providers do not have the resources to stop working and go to university.

### Spreading the word

We asked participants how they would disseminate the guidelines. Providers expressed ways to strengthen guideline dissemination: i) technology and end-user documents; ii) clear and consistent communication from stakeholders; and iii) using local champions for dissemination and implementation.

Providers referenced online (websites) and mobile technology (apps) as key tools to promote the dissemination of the guidelines as a *“summarised version of those guidelines”* (ECP, private sector). Even among older paramedics, apps seemed an attractive solution and a viable alternative to handbooks. Furthermore, providers advocated for an end-user document as a simpler, condensed “*quick reference guide*” (ILS, private sector), specific to qualifications and highlighting changes, that allows providers to see the continuum of care across all provider levels. Providers suggested the development of the guidelines could be farmed out to various institutions (like colleges or professional societies) and”*using the clinical guidelines that was put together with the evidence*, *[to] develop a flow process”* (ILS, public sector) and end-user reference book. Furthermore, an advisory committee could be established to independently review the developed end-user documents and whether they aligned to the”*spirit of the original guidelines without being prescriptive”* (ECP, private sector).

Communication was seen as a key factor in repairing the broken relationship between industry and regulators and higher education institutions:

*“Communicating with us would go a long way in repairing the relationship”* (CCA, private sector).

*“You want these protocols in place*, *it’s fine*. *You come down here*, *sit and talk to me about it”* (CCA, private sector).

Providers want a clear plan communicated to them, detailing the why, the when and the how. Providers highlighted various other suggestions for communication, stratified into existing and new methods in Table 2.

**Table 2:** Suggestions from providers: Guideline dissemination methods.

Existing methods	New (additional) methods
• Subscription emails • Standard postbox mail • Webpage • Newsletters • Roadshows/CPD	• Social media (Twitter, Facebook and YouTube) • Forums • Television and radio • Podcasts • Workshops

Providers emphasised using local champions (shift leaders, respected industry practitioners, colleges, and universities) to disseminate guideline information, build capacity and strengthen the uptake of the guidelines through leading the conversation at the coal-face level:

*“Through colleges*, *through universities*, *through even small group stations at the base levels… so it has to be introduced in a way where the guys accept it”* (ECT, private sector).

*“Through clear directive communication and a strategy part and parcel with educators and senior members in industry who would eventually… filter that information via to ALS to take it down into the industry”* (ECP, public sector).

Furthermore, providers want to be involved; they understand they also have to take responsibility for helping to disseminate and implement the guidelines:

*“Let us help and let’s get a finger in the pie and do something”* (ILS, private sector).

### Challenges and opportunities in dissemination

We asked participants what their anticipated challenges with guideline dissemination would be. Key perceived challenges included the lack of and authoritarian style of communication from EMS decision makers (specifically regulators), unintended dissemination of the guidelines to EMS, and the unexpected and large guideline document received by EMS.

The lack of consistent and clear communication from regulators regarding the guidelines, career pathways, and up-skilling has left providers confused, like “*being left in the dark”* (ILS, public sector). “*We don’t’ really understand*, *they don’t explain to us why they’re changing it*. *They’re just saying*: *‘It’s changed*, *here’s the evidence’*” (ECT, private sector).

This effect was noted by rural paramedics, where providers felt “*cut off about the big things that are happening in EMS****”*** (BAA, public sector) and felt left out due to centralisation of information and Continuous Professional Development (CPD) activities often being restricted to urban areas.

Furthermore, as the guidelines were unintentionally disseminated (leaked) to providers, a fractured message was received and caused confusion among paramedics. From that point onwards providers felt communication from regulators was a “*one-way stream of information”* (NDip, public sector) with *“no regard to what we on the ground… our feelings are or our sentiments are”* (CCA, public sector). Providers expressed a significant need to be involved in the decision-making process and to have a platform to express views (and receive feedback) so that they could understand why changes were made.

Additionally, providers did not expect to receive such a large guideline document and wanted a streamlined protocol with algorithms:

*“… a barrier would be how big the books are*, *three hundred pages*, *I’ll be honest with you*, *I’m not going to read that three hundred pages in depth”* (ECP, private sector).

### Challenges and opportunity for implementation

We asked participants what their anticipated challenges with guideline implementation would be. They expressed various challenges for guideline implementation: i) concerns that current short course training framework provides a poor educational foundation; ii) a lack of enabling upward articulation; iii) fears and apprehension about how providers are going to be up-skilled; and iv) concerns that current emergency care education systems lack capacity and resources to update or train.

Emergency care providers expressed their concern that the current short course training framework provides a poor educational foundation for providers to engage, understand and interpret the new guidelines. Providers indicated their concern that the short course education system is akin to a *“spoeg en plak”* [copy and paste] system where a *“monkey see*, *monkey do learning…”* is implemented and *“…so their desire to grow and learn and research and figure it out for themselves has been lost”* (ECP, private sector). The consequence of this system failure is presented in this private sector ECP’s thoughts below:

*“The consequence to that is that they’re not understanding how to interpret the recommendations and the projected guidelines and so they are deferring to what they’ve always known*, *which is a protocol driven approach*, *where you will do this or you won’t do that*.*”*

The above observation is reflected in the attitude of short course qualified providers as their primary concern and focus is around scopes of practice and skills:

*“Patient care*, *how to manage the drugs and all*. *That’s all I want to know”* (BAA, public sector).

The guidelines implicated various levels of providers regarding scopes of practice, by increasing scope for most, but also removing some skills from others (i.e. ETI). This created a perceived implementation barrier regarding the current educational system’s capacity to facilitate this required upskill of existing providers, whether through higher education institutions, colleges or employers.

*“How on earth are we going to educate forty thousand people on new protocols and be sure that all of them have actually upskilled and updated appropriately*?*”* (NDip, private sector).

Providers expressed concern that the current educational system lacks the capacity and resources to update or retrain the existing qualified prehospital workforce. One public sector CCA indicated he has no capacity to upskill: *“I can’t leave my job and come here for four years and hope to pass something that I’m already skilled at”*, referring to obtaining a degree just to perform intubation again. Some providers attributed the barrier not to lack of capacity but to lack of will to enable upskilling via recognition of prior learning or other streams as presented by this ECP (private sector): *“I think it’s not so much that the varsities can’t cope*, *I think that there’s a lack of will to do something constructive about it”*.

We asked participants how they would implement the guidelines. Providers expressed ways to strengthen guideline implementation: i) establishing inclusive career pathways; ii) establishing online and module training; iii) using local champions; and iv) implementation by independent makers.

The guidelines have caused considerable uncertainty within the industry and providers are unsure of where they are heading as a profession. Solutions provided are two-fold: clear communication, and providing direction to the profession regarding next steps. Emergency care providers expressed the need to know where they are heading, opportunities for upskilling (or not), implementation details, timeframes and questions answered: “*If you give us a pathway and say*: *We can up-skill you*, *we can improve you*, *you will be fine*, *you will be okay*, *I’ll go to work every day with a smile on my face*, *knowing that I’m going somewhere*” (CCA, public sector).

Providers expressed that stakeholders (specifically the HPCSA PBEC) need to take ownership of the confusion caused, “*taking charge*, *standing up*, *saying*, *‘Hey guys*, *the ball was dropped*, *you all got it this way*, *it wasn’t correct’”* (CCA, private sector), and apologise to the profession. Providers felt that stakeholders have the mandate to enable change and should be taking responsibility for implementing this successfully, starting with a clear communiqué regarding *“how it’s going to be run out [implemented]…”* (ILS, public sector) and “*what our plans are*, *this is what we intend to do and these are the time frames*” (CCA, public sector). Providers expressed that they do not understand the changes:

*“Why there is such a big difference between the previous to now*?*”* (ECT, public sector).

Providers felt strongly that training local industry champions would strengthen the implementation of the guidelines (gaining buy-in), especially among junior providers who look up to senior providers:

*“Let’s do it [train] through the ALS*, *EMS is a small industry and people who have a low qualification look up to the ALS… for instance*, *I want to be like [him] one day”* (BAA, private sector).

Providers suggested universities and colleges lead the implementation of the guidelines for new providers entering the education system whereas independent professional societies like *“EMSSA and ECSSA take on some of the training programs that would allow you to increase your scope”* (ECP, public provider). The National Department of Health, together with various stakeholders such as higher education institutions and employers (such as managers) would be involved in making the implementation a success and ensuring buy-in. Additionally, providers advocated for an online modular system, “*something where I don’t have to stop working in order to accomplish that*…” (BAA, private sector).

*“Progression*, *that is why the modular system is extremely important… the outcome is still going to be the same… whether I do it modular or whether I spend two years in the university…”* (CCA, public sector).

## Discussion

In this paper we identified a sample of prehospital end-users’ experiences and perceptions of the EMS guidelines, including key challenges and recommendations for national decision makers, in order to strengthen guideline uptake in South Africa and similar contexts. Prehospital providers’ perceptions of the CPGs for emergency care in South African were largely influenced by the contrast between expectations and the eventual perceived reality of the guidelines. Across the board, providers expected an increase in their scope of practice, most equating a larger scope to better care for patients. Providers were more concerned with the scopes of practice as opposed to the actual guideline recommendations. This was highlighted in some providers’ dependence on certain skills for professional affirmation and was especially emphasised when these skills, such as drug-facilitated intubation, were removed from advanced life support (CCA and NDip) paramedics’ scope of practice. Providers were shocked and disappointed, feeling their qualification and identity hinges on their ability to perform these skills, irrespective of (or oblivious to) the evidence of harm to patients.

Understandably, these advanced life support providers felt slighted and side-lined as they could no longer perform a skill, which they have been practicing for decades, that higher qualified ECPs can still practice using rapid sequence induction. Furthermore, we think part of the anger and disappointment expressed by providers can be attributed to the overall poor communication from industry regulators, whom providers already perceive as having a hidden agenda. However, it is still unclear what the repercussions of removing drug-facilitated intubation would be on industry service delivery, especially in the public sector, which predominantly employs CCAs and NDip paramedics.

Furthermore, we identified various solutions, supported by literature, to guide decision makers to promote the uptake of their guidelines specific to prehospital care. These included educational workshops tailored to barriers [20], development of end-user documents based on the parent CPGs [21,22], the use of industry/opinion leaders [23] and implementation by impartial local stakeholders [24]. Unexpected results and novel facilitators include creating and communicating a clear career and study pathway for providers, decision makers taking ownership of failures, using online modular training for existing providers and explaining any changes contrary to the status quo. The majority of identified facilitators revolve around education and communication, highlighting the important role of stakeholders such as emergency care colleges and universities collaborating with professional societies and regulators in strengthening guideline uptake.

As the EMS industry moves from six providers to a three-provider system (ECAs, ECTs and ECPs) [25], clear leadership is essential to transition the profession at this time. The successful dissemination and implementation of the guidelines will require careful alignment of policy and action from stakeholders that considers the perceptions, barriers and facilitators of the local end-users. This is especially relevant for providers trained outside of universities, some of whom are finding it difficult to transition from a skills-based practice (protocol guided) to an evidence-based practice. Prehospital decision makers can draw from existing South African guideline initiatives such as the South African Guideline Excellence (SAGE) group [26] and international resources [27].

As limitations, it is important to note that the results reported in this paper are those of a particular point in time and may well have changed as the guideline dissemination and implementation process continues in South Africa. Moreover, our study does not shed light on decision makers’ perspectives or other stakeholders such as prehospital managers, an important subgroup when considering guideline implementation, and one which warrants attention in future research. This study does however highlight, for the first time in South African EMS history, across all EMS qualifications, various prehospital perspectives, attitudes and issues regarding how providers view themselves, the educational system, evidence, and clinical practice. These are integral in understanding the nature of EMS and is useful for decision makers to address prehospital service delivery concerns by being able to navigate the barriers around guideline uptake in South Africa and similar contexts.

## Conclusion

In order to disseminate and implement a national emergency care guideline, decision makers should take into account the perceptions, barriers and facilitators of the local end-users. Through synthesising the perceptions of prehospital providers across South Africa, we identified the profession’s expectations, its corresponding reactions leading to the challenges, and most importantly, the collective solutions proposed by paramedics.

Decision makers, such as the National Department of Health, the South African Health Professions Council of South Africa and EMS industry leaders are essential consumers of our research and thus require targeted dissemination strategies to effect policy and practice. Further activities linked to this research include disseminating results to key decision makers and formulating feasible recommendations together with the policy makers, to enable action.

## Supporting information

S1 Table EMS qualifications in context. (DOCX)

S1 Text Interview guide. (DOCX)
