# Supplementary material for: Prehospital providers’ perspectives for clinical practice guideline implementation and dissemination: Strengthening guideline uptake in South Africa
Source: PLoS One. 2019 Jul 22;14(7):e0219761. doi: 10.1371/journal.pone.0219761 (PMC6645495; doi:10.1371/journal.pone.0219761)
Supplement: S1 Table — (DOCX) [file pone.0219761.s001.docx]

# Appendix 1

***South African emergency medical service qualifications in context***

The South African EMS has seen rapid growth over the past two decades. It developed from basic certificate courses to professional undergraduate degrees, including postgraduate Masters and PhD programmes. There are three tiers of advance life support (ALS) providers, encompassing an additional 5 qualifications:

Currently the 4-week, 9 month and 2 year National Certificate courses have been phased out, the 3-month and 3 year National Diploma courses are being phased out as industry transitions to professionalise emergency care providers away from skills based short course training programs.

| **Provider Level** | **Duration** | **Qualification Name** | **Registration Category** |
| --- | --- | --- | --- |
| Basic Life Support (BLS) | 4 weeks | Basic Ambulance Assistant (BAA) | Basic Ambulance Assistant (BAA) |
| Intermediate Life Support (ILS) | 3 months | Ambulance Emergency Assistant (AEA) | Ambulance Emergency Assistant (AEA) |
| Advanced Life Support (ALS) | 9-month certificate course | Critical Care Assistants (CCA) | Paramedic |
|  | 3-year course | National Diploma |  |
|  | 2-year course | National Diploma | Emergency Care Technicians (ECT) |
|  | 4-year course  Or  3-year National Diploma with an optional 1-year Bachelor of Technology | Professional Bachelor’s Degree | Emergency Care Practitioner (ECP) |
