# Supplementary material for: Prehospital providers’ perspectives for clinical practice guideline implementation and dissemination: Strengthening guideline uptake in South Africa
Source: PLoS One. 2019 Jul 22;14(7):e0219761. doi: 10.1371/journal.pone.0219761 (PMC6645495; doi:10.1371/journal.pone.0219761)
Supplement: S1 Text — (DOCX) [file pone.0219761.s002.docx]

# Appendix 2

**Interview Guide**

**Items to consider/check:**

Recorder and Cellphone recorder

Flipchart

Extra Informed consent forms

Focus group seating map

**Topics:**

Introduction

- Who we are

Michael and Lynn (**individual introductions: affiliation and role in study**). Michael – Principle Investigator, Lynn- Qualitative Consultant and co-investigator. We are two researchers from the Department of Global Health, Stellenbosch University, Western Cape doing research in Emergency Care. Michael’s background is in emergency care, biostatistics and epidemiology while Lynn’s background is in psychology and epidemiology. **We are very excited to be here and hear your views regarding this topic.**

- Research Objectives

The goal of this project is to **explore the perceptions of paramedics for the implementation and dissemination of the new CPGs** in SA to strengthen the uptake of the guidelines. Essentially asking what are all the **things that need to be taken into account** (like needs, barriers, solutions) by stakeholders like NDoH and the PBEC **for the successful and acceptable implementation** of the CPGs in EMS. In order to answer this question we are asking EMS providers across South Africa in focus groups like these **to share your thoughts** regarding how we can improve the acceptance and use of the new CPGs.

The two major topics we will be taking about today are the dissemination of the guidelines and the implementation of the guidelines. **Dissemination** means how one would **spread or circulate** the guidelines across South Africa**. Implementation** means putting the guidelines **into effect, using** or applying the guidelines by EMS providers.

- Rules of engagement/ group discussion

As you know this focus group is **completely voluntary**; your identities will not be used including your work affiliations. We ask that you **please turn off your phones, radios** or any other recording electronic equipment to prevent interruptions and ensure privacy. Since this is a group discussion we would like to ask that we make a group contract to protect each other’s opinions and confidentiality **[establish group contract].** This is a safe place, where you can voice yourself as you see fit, we **value everyone’s opinions** and thus please do not speak over other people and take care not to hog the conversation.

There are no right or wrong answers, we are really **just here to know your thoughts!** We are here to hear what you have to say, nothing else! You are welcome to respond to each others’ comments, even disagree, but please keep engagement **respectful**. Myself and Lynn **will guide and direct** the discussions as we have various topics to discuss. We do have limited time and will sometimes if needed carry on to the next topic. We will have a **small break** in between sessions (implementation and dissemination).

- Recording

This interview will be **audio recorded** so that we can later look again at what was said and analyse the results across South Africa. We will also be making **notes every now and then**; this are just reminders for us about the conversation. Note to participants that they **say their names** before speaking so that we can place their comments in context.

**Guideline dissemination:**

1. Please briefly introduce yourselves to the group
2. What is your experience with the Clinical Practice Guidelines (CPG)?

Probe: Knowledge, attitudes, experiences

1. Thinking back to the first time you heard about the CPGs… when and how did you hear about the EMS CPGs for the first time?
2. When you received the CPGs for the first time, what were you expecting to see?
3. We would like to talk about how we could disseminate the EMS CPGs and would like your input. What does CPGs dissemination mean to you?

Probe: What makes dissemination successful?

1. How would you like the CPGs to be disseminated and who should it be targeted towards?
2. How would you like the EMS CPGs to be shared with you?

Probe: Is there a specific format you prefer? Handbooks? Apps? Online?

Probe: Are there ways of sharing that may be inaccessible for you?

1. Who is responsible for the CPG dissemination?

*Thank you for your participation.*

*Review what was said and allow for reflections and questions*

*Break and then continue with focus group.*

**Guideline implementation:**

1. We would like to talk about how we could implement the EMS CPGs and would like your input. What does CPG implementation mean to you?

Probe: What makes implementation successful?

1. What are your thoughts on how you think the EMS CPGs can be implemented in South Africa?

*(Facilitator to write down the lists of suggestions, then mark with asterisks in follow up questions, which apply to rural or urban and public or private. Facilitator can also further probe for more discussion)*

Probe: Public or private

Probe: Urban or rural

Probe: More suggestions. What would make it successful and acceptable?

1. Do you think there are any barriers to the EMS CPG Implementation?

Probe: Examples of barriers to implementation for other past protocols

Probe: Behaviour change/clinical practice/education/resources

1. Do you think there are any facilitators to the EMS CPG Implementation?

Probe: Examples of facilitators of implementation for other past protocols

Probe: Behaviour change/clinical practice/education/resources

1. *(After questions 11 and 12, facilitator can refer back to list of implementation suggestions and identify specific barriers and facilitators to suggestions provided.)*
2. Who should be responsible for CPG implementation?
3. What does it mean to you to have a skill removed or added from your scope of practice?

Probe: What effect does that have on you? Feelings? Confidence?

*Thank you for your participation.*

*Review what was said and allow for reflections and questions*

*END*
